# An Update on the Genetics of IgA Nephropathy

**DOI:** 10.3390/jcm13010123

**Published:** 2023-12-25

**Authors:** Lin-Lin Xu, Xu-Jie Zhou, Hong Zhang

**Affiliations:** 1 Renal Division, Peking University First Hospital, Beijing 100034, China; linlinxudoctor@163.com (L.-L.X.); hongzh@bjmu.edu.cn (H.Z.); 2 Kidney Genetics Center, Peking University Institute of Nephrology, Beijing 100034, China; 3 Key Laboratory of Renal Disease, Ministry of Health of China, Beijing 100034, China; 4 Key Laboratory of Chronic Kidney Disease Prevention and Treatment, Peking University, Ministry of Education, Beijing 100034, China; 5 Research Units of Diagnosis and Treatment of Immune-Mediated Kidney Diseases, Chinese Academy of Medical Sciences, Beijing 100034, China; 6 State Key Laboratory of Vascular Homeostasis and Remodeling, Peking University, Beijing 100034, China

**Keywords:** IgA nephropathy, genetics, linkage analysis, genome-wide association study, whole genome sequencing, rare variants, epigenetics

## Abstract

Immunoglobulin A (IgA) nephropathy (IgAN), the most common form of glomerulonephritis, is one of the leading causes of end-stage kidney disease (ESKD). It is widely believed that genetic factors play a significant role in the development of IgAN. Previous studies of IgAN have provided important insights to unravel the genetic architecture of IgAN and its potential pathogenic mechanisms. The genome-wide association studies (GWASs) together have identified over 30 risk loci for IgAN, which emphasizes the importance of IgA production and regulation in the pathogenesis of IgAN. Follow-up fine-mapping studies help to elucidate the candidate causal variant and the potential pathogenic molecular pathway and provide new potential therapeutic targets. With the rapid development of next-generation sequencing technologies, linkage studies based on whole-genome sequencing (WGS)/whole-exome sequencing (WES) also identify rare variants associated with IgAN, accounting for some of the missing heritability. The complexity of pathogenesis and phenotypic variability may be better understood by integrating genetics, epigenetics, and environment. We have compiled a review summarizing the latest advancements in genetic studies on IgAN. We similarly summarized relevant studies examining the involvement of epigenetics in the pathogenesis of IgAN. Future directions and challenges in this field are also proposed.

## 1. Introduction

Immunoglobulin A (IgA) nephropathy (IgAN) is one of the most common forms of glomerulonephritis all over the world and has a range of clinical symptoms, from asymptomatic microscopic hematuria to a more severe course, including sustained proteinuria and rapid decline of renal function [1]. The pathological feature is the deposition of an IgA-dominated immune complex in the mesangial area.

The pathogenesis of disease remains unclear, but its development is driven by both genetic susceptibility and environmental risk factors [2,3,4,5,6]. Although not generally considered a Mendelian hereditary disease, marked interethnic differences [7], familial clustering [8,9,10,11], and subclinical renal abnormalities among relatives of IgAN patients [1] have indicated a strong genetic component. Genetic factors undoubtedly make a substantial contribution to the occurrence and development of the disease, with an estimated heritability of 40–50% [12,13]. Throughout the past 10–20 years, genetic studies of IgAN have been based on two basic approaches: linkage studies based on familial IgAN and case-control association studies based on sporadic IgAN. Case-control association studies can be categorized into two types: candidate-gene association studies and genome-wide association studies (GWAS). It is now widely accepted that changes in gene function can occur without changes in DNA sequences, which play a significant role in the pathogenesis of many diseases [14]. Hence, we not only update the genetic studies of IgAN but also elucidate the role of epigenetics in the development of IgAN.

## 2. Linkage Studies

Linkage study is a genetic research approach based on the principle of genetic linkage, in which genetic markers are genotyped among members of a pedigree, and then a mathematical method is used to calculate whether the genetic markers are co-segregated with the disease state in the pedigree so as to search for genetic markers that are linked to the causative genes and then locate the causative genes. The first genome-wide linkage study demonstrated linkage of IgAN to 6q22–23 (named *IGAN1*) with a LOD score (logarithm of the odds score) of 5.6 under an autosomal dominant mode of inheritance with incomplete penetrance in 24 IgAN kindreds from Italy and 6 IgAN kindreds from the United States. The LOD value is a statistical assessment that evaluates the distance between two loci on a chromosome and determines whether they are likely to be inherited together. When the LOD > 3, it is generally considered to be significant for linkage. There were approximately 60% of kindreds linked to this locus, suggesting a possible genetic heterogeneity in familial IgA nephropathy [15]. In the year 2006, Bisceglia et al. [16] performed a genome-wide linkage study in 22 new informative Italian multiplex families with IgAN, which replicated linkage to 6q22–23 (*p* = 0.01 in multipoint nonparametric analysis) and identified two suggestive linkage signals in the region 4q26–31 with a LOD score of 1.83 and 17q12-22 with a LOD score of 2.56, suggesting genetic heterogeneity among families with IgAN. In 2007, a novel IgAN susceptibility locus on chromosome 2q36 with a maximal LOD score of 3.5 was detected in a large Canadian four-generation family of German-Austrian descent with 14 affected individuals through the genome-wide linkage study [17]. At least 22 annotated and 17 nonannotated genes were identified in this region of chromosome 2q36 by querying the UCSC Human Genome Browser, including the *COL4A3* and *COL4A4* genes, which were associated with the development of Alport syndrome [18] and thin basement membrane disease [19]. Notably, the previously reported loci (6q22, 4q26–31, and 17q12–22) were not linked to IgAN in this pedigree. Similarly, in the subsequent linkage study, there was no evidence of linkage to the previously reported IgAN loci on chromosomes 6q22–23, 2q36, and 4q22–31 in a large Lebanese-Druze kindred with 38 family members [20].

Although none of the above linkage studies identified causal genes associated with IgAN, linkage studies based on different familial IgAN have identified their specific genomic region linked to IgAN, which showed the genetic heterogeneity of IgAN and the presence of different disease subtypes in pedigrees of different ethnicities.

With the rapid development of high-throughput sequencing technologies, whole-exome sequencing (WES)/whole-genome sequencing (WGS) is used to detect genetic variants on a scale of the entire human genome. The WGS/WES, combined with the linkage study, may be able to identify genetic variants underlying the loci co-segregating with IgAN. The relevant literature was searched via PubMed using the following keywords: “IgA nephropathy AND whole-exome sequencing AND pedigree” or “IgA nephropathy AND whole-genome sequencing AND pedigree” or “IgA nephropathy AND whole-exome sequencing AND families” or “IgA nephropathy AND whole-genome sequencing AND families”. There have been at least nine studies using whole-exome sequencing in familial IgAN to identify probable disease-causing mutations to date [21,22,23,24,25]. Liu et al. [21] identified six deleterious variants associated with IgAN in four genes, *MYCT1*, *DEFA4*, *ZNF543*, and *CARD8*, in 10 IgAN families. Although no causal variants were confirmed, they identified the polygenic nature of IgA nephropathy by illustrating shared co-segregating genes and variants in different families. Milillo et al. [22] identified a possible causal variant in the *SPRY2* gene co-segregating with IgAN in a Sicilian family, which can inhibit the MAPK/ERK1/2 pathway.

Cox et al. [23] found 24 variants on 23 genes co-segregating with the IgAN in 8 IgAN families by WES, which were further validated by Sanger sequencing and variant segregation analysis in an independent cohort of 240 IgAN cases and 113 healthy subjects, as well as in the 16 kindreds. These genes were interconnected in an immune-related functional network with *AKT*, *CTNNB1*, *NFKB*, *MYC*, and *UBC* as central nodes that were the key modulators of the WNT/β-catenin and PI3K/Akt pathways, which have been linked to the development of IgAN [26,27]. Stapleton et al. [25] performed WES in 10 Irish families with IgAN and identified 3 candidate variants. They found a variant of unknown significance in *COL4A3* in one family and a likely pathogenic variant in the *COL4A5* gene in another family (notably, this mutation was not present in the proband). The mutations in *COL4A3*/*COL4A5* were implicated in the pathogenesis of Alport syndrome and thin basement membrane disease (TBMD) [18,19]. The mutation in *COL4A3* had also been reported in a family diagnosed with focal segmental glomerular sclerosis [28]. Although this study did not indicate a causal relationship between variants on the *COL4A5*/*COL4A3* gene and familial IgAN, it illustrated that the mutations in the *COL4A5*/*COL4A3* gene may cause a wide spectrum of diseases, not just Alport syndrome. Previous studies have suggested that type IV collagen mutations may be associated with IgAN [17], and IgAN was observed to co-aggregate with TBMD in familial IgAN [29]. Li et al. [24] analyzed the association between the exome sequence data of 46 familial IgAN patients and *COL4A3/4/5* variants. They identified likely pathogenic *COL4A3*–*5* variants co-segregating with IgAN in 9 families, which showed that type IV collagen mutations may modify IgAN. In a recent study, Yuan et al. [30] identified diagnostic variants on the *COL4A3/4/5* genes in 31.1% of IgAN patients with thinned glomerular basement membrane (tGBM), which suggested a contribution of variants on the *COL4* gene to the tGBM phenotype in sporadic IgAN. The potential mechanism for individuals with variants in the *COL4A3*–*5* gene predisposing to IgA glomerulonephritis may be that the associated GBM thinning facilitates the emergence of IgA molecules from the glomerular capillaries and subsequent deposition in the mesangial area of the glomeruli [31]. Although possible candidate genes could be identified by WES/WGS, phenotype misspecification also limits the power and ability to precisely detect the candidate gene co-segregating with IgAN. Because the gold standard for IgAN diagnosis is an invasive renal biopsy, it is difficult to accurately identify family members as “affected” or “unaffected” due to the absence of a dependable noninvasive screening test. The development of non-invasive diagnostic biomarkers for screening family members at risk of IgAN may be needed in the future to address this issue.

## 3. Candidate-Gene Association Studies

The approach of candidate-gene association studies involves examining single-nucleotide polymorphisms (SNPs) in specific genes that are assumed to be involved in disease pathogenesis. To date, there have been over 160 candidate-gene association studies conducted for IgAN. In the early days, most candidate genes for IgAN were chosen with limited evidence of their involvement due to our lack of understanding of disease pathogenesis. The candidate genes selected for early studies were mainly the MHC class II gene, the *T-cell receptor (TCR) α* or *β chain* gene, cytokine-related genes, and renin-angiotensin system-related genes, which were mainly focused on the progression of IgAN rather than the disease susceptibility. Unfortunately, none of these studies were independently validated in other cohorts due to insufficient methodologies (poorly matched controls, inadequate variant coverage in candidate genes, and a small case-control population) and a lack of statistical power.

However, it is undeniable that there have also been studies that have made some important discoveries through candidate gene strategies. With the progressive understanding of the pathogenesis of IgAN, aberrant glycosylation of IgA1 is considered the most important pathogenic mechanism of IgA nephropathy [32,33,34,35], which provides a new logical candidate for genetic association studies. Glycosyltransferases involved in glycosylation of IgA1 (*C1GALT1*, *C1GALT1CA*, and *ST6GALNAC2*) were selected as candidate genes for analyzing their association with IgAN risk. Li et al. [36,37] proposed for the first time that the variants in the *C1GALT1* gene and *ST6GALNAC2* gene were associated with IgAN susceptibility in the Chinese population through a case-control association study in 670 IgAN patients and 494 geographically matched healthy subjects. The association between variants of the *C1GALT1* gene and IgAN susceptibility has been replicated independently in several studies based on the European and Chinese populations [38,39,40]. In addition, the interaction between *C1GALT1* variants and *ST6GALNAC2* variants in IgAN has been identified, which not only suggested the involvement of the two genes in predisposition to IgA nephropathy but also demonstrated the significance of genetic interaction for disease susceptibility [41].

## 4. Genome-Wide Association Studies

**Figure 1:**
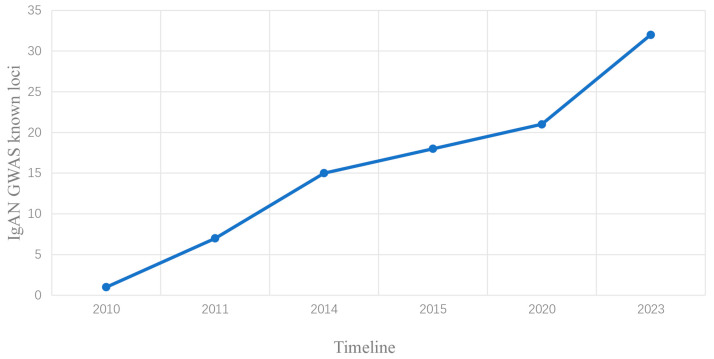
**The IgA nephropathy GWAS study timeline.** Progress in IgA nephropathy GWAS studies over the last decade or so and cumulative known risk loci at corresponding time points.

In contrast to the candidate-gene association study, the genome-wide association study (GWAS) adopts a hypothesis-free approach to identify genetic variants (e.g., single-nucleotide polymorphisms, also known as SNPs) associated with disease in a case-control population, even in the setting of significant locus heterogeneity. Population stratification can be controlled by correcting for principal components to avoid false-positive findings. In the last 15 years, genome-wide association studies (GWAS) have offered valuable insights into the genetic architecture of IgA nephropathy (Figure 1). For the purpose of reviewing the relevant literature, enter the keywords “IgA nephropathy AND genome-wide association study” in PubMed. In addition, we checked qualitative secondary sources to make sure that we had not missed any relevant studies. The first GWAS in IgAN was published in 2010 by Feehally et al. [42], with only one identified locus in HLA. Later, Gharavi et al. [43] identified three independent genome-wide significant loci in MHC, as well as a common deletion of *CFHR1* and *CFHR3* on 1q32 and a locus on 22q12. Yu et al. [44] not only confirmed the association between loci on MHC as well as 22q12 and IgAN, but also identified associations at 17p13 and 8p23, which encoded tumor necrosis factor (*TNFSF13*) and α-defensin (*DEFA*), respectively. In 2014, a trans-ethnic GWAS meta-analysis by Kiryluk et al. [4] identified nearly 20 independent risk alleles for IgA nephropathy in predominantly European and East Asian ancestry, which explained approximately 7% of total disease risk. The proteins encoded by these loci are involved in the innate and acquired immunity, complement pathway, and maintenance of the intestinal mucosal barrier and intestinal network of IgA production, which provides insights into the pathogenic mechanisms of IgA nephropathy. Due to the space limitations, the specific findings of previous GWAS studies can be detailed in Table 1 and other expert reviews [6,45].

In 2020, Li et al. [48] identified three novel loci associated with IgAN (rs6427389 on 1q23.1, rs6942325 on 6p25.3, and rs2240335 on 1p36.13) by performing a three-stage meta-analysis of four previously published GWAS studies of IgAN [4,43,44,46] in 10,546 cases and 21,871 healthy controls, implicating *FCRL3*, *DUSP22/IRF4*, and *PADI4* as novel candidate susceptibility genes for IgAN. They also illustrated the ethnic heterogeneity of seven loci out of 24 confirmed risk variants between Chinese and European ancestry, which may explain the differences in susceptibility between European and Chinese ancestry. These risk loci identified by GWASs were common SNPs with a minor allele frequency of >5% in the population, which usually have a small effect size and are located in the non-coding regions. Few previous studies have systematically assessed the rare protein-coding variant in IgAN. Entering the keywords “IgA nephropathy AND exome association study” in PubMed, we found that only two studies have investigated the susceptibility of coding region variants to sporadic IgAN through the exome chip-based case-control association study in the last five years. Zhou et al. [49] performed an exome chip-based association study in 601 IgAN patients and 4076 healthy controls of Han Chinese ancestry and replicated their findings in an independent cohort of 2762 cases and 5803 healthy controls. They identified one novel genome-wide significant variant in the HLA region (*GABBR1*) and three suggestive non-HLA variants in the *FBXL21*, *CCR6*, and *STAT3* genes. Significantly, since this exome chip were designed on the basis of sequencing data from a multiracial population, many rare Asian-specific variants will be lost. Recently, Li et al. [50] performed a three-stage exome chip-based association study of coding variants in 31,753 Han Chinese participants (8529 IgAN patients and 23,224 healthy individuals) and identified a novel rare nonsynonymous risk variant in *VEGFA* and a common nonsynonymous risk variant in *PKD1L3*.

Very recently, the up-to-date largest GWAS meta-analysis from 17 international case-control cohorts has identified 30 genome-wide significant risk loci of IgAN, including 16 novel loci in 10,146 IgAN patients and 28,751 healthy controls, which explained 11% of overall disease risk [51]. Using tissue- and cell-type-specific FUN-LDA scores, the researchers partitioned SNP-based heritability to map the most likely causal tissues and cell types. They found the most significant heritability enrichments in blood immune cells and gastrointestinal mucosa cells. In addition, other analytical approaches have also indicated that extrarenal tissues may play a causal role in IgAN. Specifically, hematopoietic, immune, and gastrointestinal tissues are the most likely to harbor causal cell types. In pathway enrichment analysis based on genome-wide significant non-HLA loci, the enrichments mainly focused on innate and adaptive immunity, with the most significant enrichment in “cytokine-cytokine receptor interactions”. A protein-protein interactions network constructed by proteins encoded by candidate genes within the genome-wide significant loci also indicated the significance of the immune responses as well as chemokine and cytokine-mediated signaling. They identified enrichment in soluble ligand-receptor pairs, such as APRIL-TACI encoded by *TNFSF12/13* and *TNFRSF13B*, and several IL6-related cytokine-receptor pairs (IL6-IL6ST, LIF-LIFR/IL6ST, and OSM-OSM/LIFR/IL6ST), which were associated with IgA homeostasis and the production of the galactose-deficient IgA1 [55,56,57,58]. These results emphasized the importance of IgA production and regulation in the pathogenesis of IgAN, where the kidney was more likely to be the victim. The identification of cytokine ligand-receptor pairs likewise provides potential targets for further specific therapy in IgAN. They also performed genome-wide genetic correlation analyses associated with immune, infectious, and cardiometabolic traits to explore whether IgA nephropathy shared a genetic architecture with other traits. It was discovered that the most significant positive genetic correlations with IgA nephropathy were pneumonia (*r_g_* = 0.26, *p* = 9.0 × 10^−3^) and urinary tract infection (*r_g_* = 0.25, *p* = 2.1 × 10^−3^). Significantly, they also found a strong positive association of the IgAN 15-SNP genetic risk score derived from the previous study [4] with local pathogen diversity (*r* = 0.61, *p* = 6.0 × 10^−7^). Additionally, Zhou et al. [49] identified novel suggestive genes (*CCR6*, *STAT3*, *TGFBI*, *GABBR1*, and *CFB*) associated with IgAN through an exome chip-based association study, which suggested multiple pathways related to immune response, and the most significant KEGG pathway was *Staphylococcus aureus* infection. Some of the genetic factors identified in these recent studies have been linked to mechanisms of defense against infection. Therefore, follow-up studies are needed to investigate how infection triggers mucosal immunity, causing IgAN, and identify the specific pathogenic bacteria or viruses. 

Another effective GWAS method involves identifying genetic variants associated with disease-related quantitative endophenotypes, such as serum galactose-deficient IgA1 level, which has been reproducibly associated with IgAN and its progression, and it has a high degree of heritability in family-based studies, ranging from 40% to 80% [13,59]. Genetic studies of complex diseases can use quantitative endophenotypes as the outcome, a measurable intermediate phenotype of the diseases, as they could reflect underlying pathogenic processes and provide greater statistical power to identify genotypic correlations [60]. Both previous GWASs of serum Gd-IgA1 levels identified two genome-wide significant signals on the *C1GALT1* and *C1GALTC1* genes in European and East Asian ancestry [52,53]. Nevertheless, neither of the identified variants has been associated with susceptibility to IgAN. Additionally, these two loci explained about 7% of the variability in serum Gd-IgA1 levels in Europeans, but only 2% in East Asians [53]. A recent GWAS of the serum Gd-IgA1 levels by Wang et al. [54] offered some new insights into the genetic factors of serum Gd-IgA1 levels and their genetic link with IgAN susceptibility in the Chinese population. They confirmed the association between *C1GALT1* and serum Gd-IgA1 levels, but with a different lead SNP (rs10238682), explaining 3.7% of variability in serum Gd-IgA1, which was significantly higher than previously reported loci (rs13226913) explaining only 0.9% of variability in the Chinese ancestry. In addition, a novel locus for serum Gd-IgA1 levels at *GALNT12* has been identified, which explained about 3.5% of the variance. These two loci had genetic associations with IgAN susceptibility, except that the correlations did not survive Bonferroni correction for multiple comparisons. The additive interaction analysis revealed an interactive effect between *GALNT12* and *C1GALT1* in both serum Gd-IgA1 levels and IgAN risk. By adding the interaction term, the total variance explained jointly by the *C1GALT1* and *GALNT12* loci increased from 6.6% to 7.2%.

In summary, GWAS has successfully uncovered the genetic architecture of IgA nephropathy. However, the 30 genome-wide significant risk loci identified by the latest GWAS of IgAN with the largest sample size only explained 11% of total disease risk, which suggested that there were still a large number of genetic variants remaining to be discovered. This phenomenon is not unique to the field of IgAN, which is an inherent limitation of GWAS. Firstly, GWASs can only identify common variants associated with the disease, which usually have relatively small or moderate effect sizes. Secondly, most of the loci identified through GWASs are situated in the non-coding region, and many are far from the discovered genes. Thirdly, GWASs are not always replicable across different studies or populations. Lastly, previous GWAS DNA chips were fixed and had limited coverage of single nucleotide polymorphisms (SNPs) in exon and promoter regions. Additionally, research on the impact of rare variants is still lacking, which could be addressed by using a whole-genome sequencing approach in large cohorts. WGS technology has successfully identified rare variants with stronger effects in complex diseases like psychiatric disorders [61] and cardiovascular diseases [62,63], which greatly improved heritability. It is expected that population-based genetic association studies based on WGS technology can be used to identify variants with larger effects associated with IgAN susceptibility.

## 5. Post-GWAS Studies in IgAN

In the last 15 years, genome-wide association studies have successfully identified more than 30 genetic loci associated with IgAN risk [4,42,43,44,46,48,51]. However, these variants are not necessarily causal variants due to the linkage disequilibrium. In addition, most of these variants are in non-coding regions of the genome, and it is difficult to translate these genetic association results into interpretable biological mechanisms. Hence, it is significant to evaluate the GWAS loci by fine-mapping and functional studies to identify the underlying causal genes and relevant causal tissues or cell types. Previous expert reviews have discussed the post-GWAS fine-mapping studies that involved the HLA region on chromosome 6p21, the locus containing the Complement Factor H (*CFH*) gene along with five genes encoding FH-related peptides on chromosome 1q32, and the locus encoding α-defensin 1 and 3 on chromosome 8p23 [6,45]. The fine-mapping of the *CFH* gene and its neighboring regions identified the deletion of the *CFHR3* and *CFHR1* genes (CFHR3,1Δ) as the most likely causal protective variation, which indicated the role of the dysregulated activity of the alternative complement pathway in the pathogenesis of IgAN [64]. The fine-mapping study of the 8p23 locus successfully identified that higher copy numbers of the *DEFA1* and *DEFA3* genes were protective from IgAN, which explained 4.96% of the disease risk in Chinese [65]. Next, we discuss more refined and standardized fine-mapping studies associated with GWAS loci in the last five years, which provide additional insights into understanding the causative genes and associated mechanisms of IgAN.

The *MTMR3*/*HORMAD2*/*LIF*/*OSM* region on chromosome 22q12.2 has now been identified by at least eight IgAN-related GWASs, which has been reported to be associated with an increasing risk of tonsillectomy and upper respiratory infections [66,67], suggesting that the gene(s) underlying this region may play a significant role in mucosal IgA responses to infectious pathogens. *LIF*/*OSM* encoding two IL-6-related cytokines were involved in mucosal immunity and inflammation, which has also been reported to be associated with the production of IgA [68]. Although both *LIF* and *OSM* are potential candidate genes, the specific causal variant and its target gene for this locus are still unresolved. Wang et al. [69] analyzed the association of variants located in and around the *MTMR3*/*HORMAD2*/*LIF*/*OSM* region with IgAN susceptibility and identified rs4823074 as the most significant variant in this region, which was also the lead SNP of this region in the more recent IgAN-related GWAS meta-analysis with the largest sample sizes [51]. Although rs4823074 is an intronic variant in *HORMAD2*, *MTMR3*, encoding a member of the myotubularin family that has phosphoinositide 3-protein tyrosine phosphatase activity, it was identified as a likely candidate gene through comprehensive eQTL and gene expression analyses based on different strategies. A causal effect of serum IgA levels on IgAN risk at the locus has been identified through colocalization and Mendelian randomization analyses. And these results suggested that this region may affect serum IgA levels by increasing MTMR3 expression, which further contributed to the development of IgAN. They demonstrated that the MTMR3 could increase the production of IgA1 by mediating activation of the TLR9 pathway in vitro experiments, which was further confirmed in vivo CRISPR-Cas9 animal studies. This study not only highlights the role of *MTMR3* in the production of IgA1 as well as its susceptibility to IgAN, but also provides a new potential target for the treatment of IgAN.

**Figure 2:**
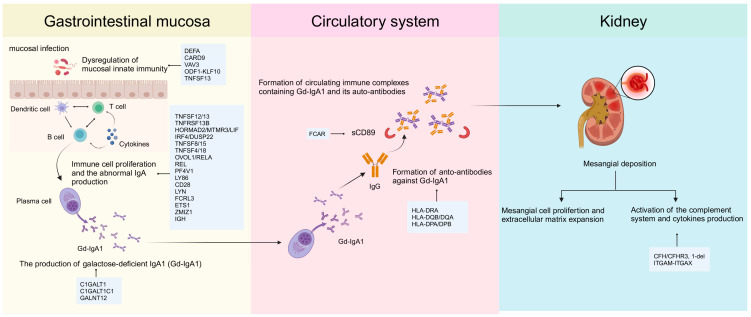
**The four-hit hypothesis and possible candidate genes involved in the corresponding process.** The four-hit hypothesis of IgA nephropathy: Firstly, the level of IgA1-bearing galactose-deficient O-glycans (Gd-IgA1) is increased in the circulation of patients with IgAN. Then, these Gd-IgA1 are recognized as autoantigens by antiglycan autoantibodies, leading to the formation of immune complexes that accumulate in the glomerular mesangium. Finally, these immune complexes deposit in the glomerular mesangial area, activate mesangial cells, induce proliferation, and promote the production of extracellular matrix, cytokines, and chemokines, leading to renal injury. A light blue color highlights candidate genes based on the GWAS findings that may be involved in the corresponding process. Created with BioRender.com (https://www.biorender.com/ (accessed on 12 December 2023)).

Indeed, many GWAS causal variants have been thought to operate by regulating gene expression in specific cell types through regulatory elements [70,71,72]. Hence, it is of significance to integrate functional genomics datasets and follow-up functional experimental studies of GWAS loci to reveal the true pathogenic variants and their underlying genetic pathogenic mechanisms. In this review, we simply queried the Open Target Genetics database [73] for 30 genome-wide significant SNPs discovered by the recent largest GWAS study of IgAN to get evidence of functional genomics data supporting their corresponding candidate genes (Table 2). At the same time, we mapped these candidate genes identified based on the largest sample size GWAS study to the potential pathogenic processes in which they may be involved (Figure 2).

## 6. Epigenetic Studies of IgAN

Epigenetic factors also play an important role in the pathogenesis of IgAN. Epigenetic regulation modulates gene transcription without changing DNA sequences [75]. The primary mechanisms involve DNA methylation, histone covalent modification, chromatin remodeling, and RNA interference. The interactions of genetics, epigenetics, and environmental factors altogether contribute to the development of IgAN.

Studies have demonstrated that changes in DNA methylation might differentiate IgAN from healthy individuals. Sallustio et al. [76] performed a genome-wide screening for DNA methylation in CD4^+^ T cells from IgAN cases and healthy controls and found 281 CpG sites differentially methylated in IgAN cases compared with healthy controls, which were mostly overrepresented in promoters of genes involved in T cell signaling. They identified aberrant methylation in the *TRIM27*, *DUSP3*, and *VTRNA2-1* (miR-886 precursor) regions influencing the expression of these genes in IgAN patients, which led to the reduced TCR signal strength of the CD4^+^ T cells. They revealed an abnormal CD4^+^ T cell response, which may explain the T helper cell imbalance in the Th1 subtype. An epigenome-wide association study based on B cell-specific samples from 92 IgAN patients and 92 healthy controls identified three genome-wide significant CpGs corresponding to *PCDH17*, *TERT*, and *WDE82* genes and three in the intergenic regions. A weighted gene co-methylation network has been constructed, and an additional IgAN-associated gene module containing 72 significant CpGs was identified, which included *GALNT6*, *IQSEC1*, *CDC16*, and *SYS1* that are involved in the pathway related to tubular atrophy/interstitial fibrosis of IgAN [77].

MicroRNAs (miRNAs) are short, non-protein-coding RNAs consisting of 20–25 nucleotides. They work by binding to the 3′-untranslated region (3′-UTR) of the target mRNAs, thereby repressing gene expression in the post-transcriptional process. Because the regulation of miRNA-mediated gene expression is one of the epigenetic mechanisms [78], there have been numerous studies on various miRNAs in IgAN [79,80]. There have been many previous studies that have identified some miRNAs associated with the occurrence and development of IgAN, which may play an important role in the production of the Gd-IgA1 [81,82,83,84], immunoregulatory disorder [83,84,85,86,87], renal interstitial fibrosis [88,89,90], and inflammatory response of IgAN [91,92,93]. The levels of miR-23b-3p (miR-23b) were decreased in the kidney biopsies and serum of IgAN patients compared with healthy controls. The miR-23b^-/-^ mice developed an IgAN-like phenotype characterized by the development of albuminuria, impairment of kidney function, mesangial IgA, and C3 deposition [94]. The miR-150-5p was not only associated with IgAN [95], but also associated with the progression of IgAN [90]. In situ hybridization revealed that the location of miR-150-5p in the kidney, which was mainly present in the kidneys of IgAN progressors, was associated with mononuclear infiltrates in the tubulointerstitial region, particularly in lymphoid nodules, largely located in areas of scarred cortex and atrophic tubules.

## 7. Future Perspectives and Challenges

Genetic studies of IgAN have supported our understanding of the genetic architecture of IgAN, from initial linkage and candidate-gene studies to current large genome-wide association studies. Although GWAS studies have generated a list of IgAN risk loci, these susceptibility loci explain only a small fraction of the disease heritability. Currently, rare variants are thought to have a larger effect on complex diseases, and structural variants are believed to contribute to the development of complex diseases [96,97,98]. Therefore, the integration of common and rare variants will lead to a broader understanding of the genetic architecture of IgAN and to the identification of causal genes and biological processes. As the cost of high-throughput sequencing decreases and statistical methods for analyzing sequencing results improve, we can mine the genome deeply for new loci and assess the contribution of low-frequency/rare variants and structural variants to pathogenesis.

More importantly, we need to explore whether and how risky candidate genes are involved in the development of IgAN. Since IgAN is a highly heterogeneous complex disease, including clinical phenotypes, pathological features, and prognosis, this contributes to the difficulty in identifying genetic risk factors. Hence, we also need to identify genetic factors that influence clinical sub-phenotypes and the risk of progression to end-stage renal disease. In addition, patients at high risk of progression may benefit from glucocorticoids [99], but they can cause serious infectious complications. Therefore, it is important to identify which patients should receive glucocorticoid therapy. Pharmacogenomic studies using genetics to predict treatment responses may solve this question [100,101]. As molecular changes typically precede clinical manifestations, it is of great significance to integrate these risk loci with multi-omics data (e.g., transcriptomics, epigenomics, proteomics, and microbiomics) to comprehend the role of genetic variants in the pathogenesis of IgAN. Additionally, multi-omics longitudinal measurements combined with clinical sub-phenotypes in prospective cohort or randomized clinical trials could identify novel biomarkers that could be used for clinical diagnosis as well as patient stratification and distinguish responders from non-responders.

Another challenge is to identify the causal associations based on these observational data from the cohorts of IgAN patients. We may move from analyses at the level of omics data to analyses at the level of SNPs in omics data (xQLTs). For example, in analyzing the transcriptome data, we could move from analyses at the levels of transcripts to analyses of expression quantitative trait locus (eQTL). This helps to elucidate the molecular mechanisms underlying the development of IgAN and contribute to its translation into clinical applications, such as genetic screening, diagnosis, risk stratification, and the development of related targeted therapeutic drugs.

In conclusion, the major aim of genomics research is to accurately predict disease risk and inform clinical decision-making. It is important to translate the findings of genetics into clinical practice and personal medicine.

## Figures and Tables

**Table 1:** Summary of GWAS loci associated with IgAN or serum Gd-IgA1 levels.

Study	Published Date	Ancestry	GWAS Population	Genome-Wide Significant Loci (Candidate Gene)
Susceptibility to IgA nephropathy				
Feehally et al. [42]	2010	European ancestry	244 cases and 4980 healthy controls	6p21 (HLA)
Gharavi et al. [43]	2011	Chinese and European ancestry	3144 cases and 2822 healthy controls	6p21 (HL-DQB1/DRB1; PSMB9/TAP1; HLA-DPA1/DPB2), 1q32 (CFHR3/R1), 22q12 (HORMAD2)
Yu et al. [44]	2011	Chinese ancestry	1434 cases and 4270 healthy controls	6p21 (HLA), 8p23 (DEFAs), 17p13 (TNFSF13), 22q12 (MTMR3)
Kiryluk et al. [4]	2014	European and East Asian ancestry	7658 cases and 12,954 healthy controls	6p21 (HLA-DQ-HLA-DR; TAP1-PSMB8; HLA-DP),1p13 (VAV3), 1q32 (CFHR3-CFHR1 deletion), 8p23 (DEFAs), 9q34 (CARD9), 16p11 (ITGAM-ITGAX), 17p13 (TNFSF13), 22q12 (HORMAD2)
Li et al. [46]	2015	Chinese ancestry	1434 cases and 10,661 healthy controls	6p21 (HLA), 3q27 (ST6GAL1), 8p23 (DEFA), 8q22 (ODF1-KLF10), 11p11 (ACCS), 16p11 (ITGAX-ITGAM), 22q12 (HORMAD2)
Jeong et al. [47]	2019	Korean ancestry	188 cases and 455 healthy controls	10p15 (ANKRD16) (*p* = 0.0045)
Li et al. [48]	2020	Chinese and European ancestry	2628 cases and 11,563 healthy controls	6p21 (HLA), 1q23 (FCRL3), 1p36 (PADI4), 6p25 (DUSP22/IRF4), 8p23 (DEFA), 16p11 (ITGAX-ITGAM), 17p13 (TNFSF12-TNFSF13), 22q12 (MTMR3/HORMAD2)
Zhou et al. [49]	2021	Chinese ancestry	601 cases and 4076 healthy controls	6p21 (GABBR1), suggestive genes (TGFB1, CCR6, STAT3, CFB)
Li et al. [50]	2023	Chinese ancestry	2378 cases and 15,642 healthy controls	6p21 (HLA), 6p21.1 (VEGFA), 16q22.2 (PKD1L3), 17p13 (TNFSF13)
Kiryluk et al. [51]	2023	European and East Asian ancestry	10,146 cases and 28,751 healthy controls	6p21 (HLA), 8 known non-HLA loci (CFH, FCRL3, IRF4/DUSP22, DEFA1/4, CARD9, ITGAM/ITGAX, TNFSF13/12, MTMR3/HORMAD2/LIF/OSM), 16 new non-HLA loci (TNFSF4/18, CD28, REL, PF4V1, LY86, LYN, ANXA13, TNFSF8/15, ZMIZ1, REEP3, OVOL1/RELA, ETS1, IGH, IRF8, TNFRSF13B, and FCAR), CCR6 (only in the East Asian cohorts)
**Serum Gd-IgA1 levels**				
Gale et al. [52]	2017	European and Chinese ancestry	513 subjects	7p21 (C1GALT1)
Kiryluk et al. [53]	2017	European and East Asian ancestry	2633 subjects	7p21 (C1GALT1), Xq24 (C1GALT1C1)
Wang et al. [54]	2021	Chinese ancestry	1127 patients with IgAN	7p22 (C1GALT1), 9q22 (GALNT12)

**Table 2:** The functional genomic data for 30 recently discovered genome-wide significant SNPs is in the Open Target Genetics database.

Chr.	Position (hg19)	SNP	Candidate Gene	pQTL	sQTL	eQTL	PCHi-C (Javierre, 2016)	VEP (Ensembl)
1	157542162	rs849815	*FCRL3*					downstream gene variant
1	173146357	rs4916312	*TNFSF4*					upstream gene variant
1	196603302	rs12029571	*F13B*					intergenic variant
1	196686918	rs6677604	*CFH*					intron variant
2	61092678	rs842638	*PUS10*					intron variant
2	204584759	rs3769684	*CD28*					intron variant
4	74725320	rs6828610	*PF4V1*					regulatory region variant
6	249571	rs12201499	*IRF4*					intergenic variant
6	7214676	rs12530084	*RREB1*					non coding transcript exon variant
6	32389305	rs9268557	*HLA-DQA2*					intergenic variant
6	32599999	rs9272105	*HLA-DQA*					intron variant
6	32667829	rs9275355	*HLA-DQA2*					intergenic variant
6	32681631	rs9275596	*HLA-DQA2*					upstream gene variant
6	33074288	rs3128927	*HLA-DPB1*					intron variant
8	6808722	rs2075836	*DEFA3*					intron variant
8	56852496	rs75413466	*LYN*					intron variant
8	124765474	rs34354351	*FAM91A1*					intergenic variant
9	117643362	rs13300483	*TNFSF8*					intergenic variant
9	139266496	rs4077515	*CARD9*					missense variant
10	65363048	rs57917667	*NRBF2*					intron variant
10	81043743	rs1108618	*ZMIZ1*					intron variant
11	65555524	rs10896045	*CFL1*					intron variant
11	128487069	rs7121743	*ETS1*					intron variant
14	107222014	rs751081288	*IGH*					upstream gene variant
16	31357760	rs11150612	*ITGAX*					intergenic variant
16	86017715	rs1879210	*IRF8*					intron variant
17	7462969	rs3803800	*TNFSF13*					missense variant
17	16851450	rs57382045	*COPS3*					intron variant
19	55397217	rs1865097	*FCAR*					intron variant
22	30512478	rs4823074	*ASCC2*					intron variant

Note: SNP, single-nucleotide polymorphism; pQTL, protein quantitative trait loci; sQTL, splicing quantitative trait loci; eQTL, expression quantitative trait loci; PCHi-C, Promoter Capture Hi-C. The table above shows the results of functional genomic data in the Open Target Genetics database based on the 30 genome-wide significant variants identified in the GWAS study of IgA nephropathy with the largest sample size [51]. The most likely candidate gene for this variant were selected based on the custom V2G score from this database. The V2G scores were intended as a way to rank genes based on all available functional data. The higher the V2G score, the more evidence there was for a functional association. The blue color of the cell indicated evidence of a corresponding functional association (pQTL, sQTL, eQTL, and PCHi-C) of the variant with the candidate gene. The PCHi-C data were obtained from Javierre et al. [74]. The Variant Effect Predictor (VEP) was the most severe coding-sequence consequence of Ensembl’s Variant Effect Predictor. See the website (https://genetics.opentargets.org/ (accessed on 12 December 2023)) for specific descriptions and data sources.

## Data Availability

No new data were created or analyzed in this study. Data sharing is not applicable to this article.
